# Analgesia for Thoracic Surgery: Does Intraoperative Methadone Deserve a Place?

**DOI:** 10.1111/ans.70306

**Published:** 2025-08-21

**Authors:** Eugene Constantine Lai, William Thomas Birkett, Luxmana Jeganathan, Ashley St John, Anurag Vijay, Walston Reginald Martis

**Affiliations:** ^1^ Department of Anaesthesia and Perioperative Medicine Monash Health Melbourne Victoria Australia; ^2^ Department of Anaesthesia, Perioperative and Pain Medicine Peter MacCallum Cancer Centre Melbourne Victoria Australia; ^3^ Department of Critical Care University of Melbourne Melbourne Victoria Australia

## Abstract

Intraoperative methadone: potential benefits and considerations for thoracic surgery analgesia.

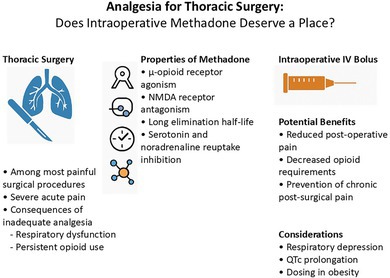

**Abbreviations**
IV: intravenous
NMDA: N‐methyl‐D‐aspartate
QTc: corrected QT interval

Thoracic surgery, whether performed through open thoracotomy or minimally invasive video‐assisted thoracoscopic surgery (VATS), remains among the most painful of all surgical interventions [1]. Despite ongoing advances in multimodal analgesia and enhanced recovery protocols, a substantial proportion of patients continue to experience significant postoperative pain and an elevated risk of developing chronic post‐surgical pain (CPSP) [2]. The acute pain associated with thoracic surgery is often severe, with detrimental effects on respiratory function, mobilization, and recovery. Inadequate pain control may contribute to prolonged hospitalization and increase persistent opioid use and its associated harms [3]. Beyond individual patient impact, CPSP carries considerable societal costs, including increased healthcare utilization, reduced workforce participation, and long‐term disability [4]. Such consequences highlight the importance of effective analgesic strategies both immediately postoperatively and in reducing the longer‐term burden of pain.

Intraoperative intravenous (IV) methadone is a promising yet underutilized intervention that may address many of these challenges. It has a unique pharmacological profile combining μ‐opioid receptor agonism, N‐methyl‐D‐aspartate (NMDA) receptor antagonism, and a prolonged half‐life. Methadone can offer a safe, effective, and affordable strategy to improve analgesia and potentially reduce opioid requirements and healthcare resource utilization in thoracic surgery [5]. An expanding body of evidence supports its use in major surgical procedures, demonstrating benefits in pain control, patient satisfaction, and reduced postoperative opioid consumption [3]. However, there is a requirement for careful implementation, including clear protocols, clinician education, and robust monitoring to mitigate risks such as delayed respiratory depression and QT interval prolongation [6].

Methadone has a long elimination half‐life of 24–36 h, which provides sustained analgesia extending beyond the intraoperative period [5]. Unlike short‐acting opioids, methadone contributes meaningfully to early postoperative pain control, reducing or eliminating the need for patient‐controlled analgesia (PCA), particularly in the first 24 h. The NMDA antagonism may reduce central sensitization and chronic post‐surgical pain, while serotonin and noradrenaline reuptake inhibition may enhance analgesic and mood‐modulating effects [7].

These properties are highly relevant in thoracic surgery. Surgical trauma, rib spreading, and intercostal nerve injury produce severe, prolonged pain. Regional techniques such as thoracic epidural or paravertebral block can provide excellent analgesia but are not always feasible [1]. Even with optimal regional blockade, breakthrough pain remains common. Methadone serves as a valuable adjunct or alternative when regional anesthesia is contraindicated.

Evidence for intraoperative methadone in painful surgeries, such as cardiothoracic procedures, is expanding. A systematic review by Lobova et al. demonstrated that intraoperative methadone (0.1–0.3 mg/kg) significantly reduced acute postoperative pain and opioid consumption without increasing adverse events in cardiac surgery [8]. Edwards et al. similarly supported its role in cardiac surgery, citing prolonged analgesia, reduced reliance on short‐acting opioids, and enhanced recovery [9]. Murphy et al. established that intraoperative methadone improved postoperative pain control, reduced opioid requirements, and improved satisfaction in both complex spinal and spinal fusion surgery [5, 7]. Boesl et al. further demonstrated benefits in cytoreductive surgery with hyperthermic intraperitoneal chemotherapy, where methadone reduced opioid consumption and shortened hospital stay compared to epidural analgesia [10]. A meta‐analysis by Schmidt et al., encompassing thoracic and other major surgeries, confirmed that intraoperative methadone improves analgesia and reduces opioid requirements without significantly increasing serious adverse events [3]. While large randomized controlled trials in thoracic surgery remain lacking, the shared pathophysiological pain mechanisms between thoracic and cardiac surgery allow cautious extrapolation of benefits.

In practice, intraoperative methadone is typically administered as a single IV bolus of 0.1–0.2 mg/kg after induction of anesthesia. This ensures peak plasma concentrations during surgery while providing sustained analgesia. Dosing is adjusted for age, comorbidities, and opioid tolerance. Close postoperative monitoring in the Post‐Anaesthetic Care Unit (PACU) remains essential. While most studies report no significant increase in respiratory depression compared to conventional opioids, mild respiratory events such as transient hypoventilation or oxygen desaturation may occur, particularly in opioid‐naïve or higher‐risk patients [6].

Dosing in patients with obesity warrants caution. There is limited data on methadone pharmacokinetics in obesity. Dosing by total body weight may risk overdose, given the poor vascularity of adipose tissue. The Specialists in Obesity and Bariatric Anaesthesia Society (SOBA‐UK) guidelines recommend dosing parenteral opioids such as morphine and fentanyl by lean body weight (LBW) in obese patients [11]. In the absence of robust data specific to methadone, LBW‐based dosing may provide a pragmatic and safer approach, especially given the increased incidence of sleep‐disordered breathing in this population [11].

Methadone integrates well with multimodal analgesia protocols, combining effectively with regional techniques, non‐opioid analgesics, and adjuncts like dexmedetomidine or ketamine. In our institutional experience, methadone has proven valuable for VATS resections where regional techniques may be limited due to anticoagulation or surgical considerations. Potential risks include respiratory depression, which, when dosing and monitoring are appropriate, does not appear more frequent than with conventional opioids [6]. QTc prolongation is a theoretical concern; however, single intraoperative doses have not been associated with clinically significant arrhythmias [7]. Caution remains advisable for patients with known prolonged QTc or on QT‐prolonging agents. Interindividual variability in methadone metabolism is another consideration, though standard dosing achieves predictable levels in most patients [3].

Beyond clinical outcomes, intraoperative methadone may offer economic benefits. By reducing reliance on PCA and improving early mobilization, it may shorten hospital stays. Rajkovic et al. demonstrated reduced length of stay for spinal surgery patients receiving methadone, further improved with adjunctive ketamine [12]. While the potential for earlier high‐dependency unit (HDU) discharge remains speculative, preventing CPSP offers a more compelling cost–benefit argument, given its substantial long‐term healthcare costs and opioid‐related morbidity. Murphy et al. provide early evidence supporting the role of methadone in CPSP prevention, meriting further investigation [2].

In summary, intraoperative IV methadone offers a safe, effective, and affordable strategy to enhance perioperative analgesia in thoracic surgery. Its unique pharmacological properties, sustained analgesic profile, and growing evidence base support broader adoption. While not a replacement for comprehensive multimodal strategies, methadone is a powerful adjunct addressing limitations of current practice. Further work on dosing protocols, especially in obesity, and health‐economic outcomes will strengthen its role. Nonetheless, given the significant burden of pain and opioid morbidity in thoracic surgery, incorporating intraoperative methadone represents a pragmatic step toward improved patient outcomes.

## Conflicts of Interest

The authors declare no conflicts of interest.

## Data Availability

Data sharing not applicable to this article as no datasets were generated or analysed during the current study.
